# Automated CT-Based Quantification of Pulmonary Fibrosis Using Deep Learning-Based Lung Segmentation

**DOI:** 10.3390/diagnostics16182907

**Published:** 2026-09-09

**Authors:** Wen-Chien Cheng, Wei-Chih Liao, Chia-Hung Chen, Chih-Yen Tu, Zhi-Ren Tsai, Jeffrey J. P. Tsai

**Affiliations:** 1 Department of Medical Research, China Medical University Hospital, Taichung 404327, Taiwan; 2 Division of Pulmonary and Critical Care, Department of Internal Medicine, China Medical University Hospital, Taichung 404327, Taiwan; 3 School of Medicine, College of Medicine, China Medical University, Taichung 406040, Taiwan; 4 Department of Life Science, National Chung Hsing University, Taichung 402204, Taiwan; 5 Doctoral Program in Translational Medicine, National Chung Hsing University, Taichung 402204, Taiwan; 6 Rong Hsing Research Center for Translational Medicine, National Chung Hsing University, Taichung 402204, Taiwan; 7 Department of Computer Science & Information Engineering, Asia University, Taichung 413305, Taiwan; 8 Center for Precision Medicine Research, Asia University, Taichung 413305, Taiwan; 9 Department of Bioinformatics and Medical Engineering, Asia University, Taichung 413305, Taiwan

**Keywords:** idiopathic pulmonary fibrosis, computed tomography, fibrosis quantification, deep learning, lung segmentation

## Abstract

**Background/Objectives:** To develop and evaluate an automated CT-based framework for the quantitative assessment of fibrotic interstitial lung disease (ILD), including idiopathic pulmonary fibrosis (IPF), using a standardised six-level anatomical protocol and deep-learning lung segmentation. **Methods:** The segmentation dataset comprised 3315 manually annotated development slices from 92 patients and a non-overlapping internal holdout of 845 slices from 5 patients. A separate 100-study localisation/scoring set yielded a 97-patient agreement cohort (84 IPF, 13 other ILD; 1164 per-level, per-lung observations) after three DICOM-conversion exclusions. YOLO11n-seg masks underwent vessel- and structure-removal fibrosis detection. The radial spatial score was compared with a non-blind expert-adjudicated reference; the per-level Fibrosis Index was an auxiliary read-out. **Results:** On the five-patient internal segmentation holdout, mean intersection over union (mIoU) was 0.926 ± 0.017; the in-sample development value was approximately 0.95. Model-only latency was 73.8 ± 9.7 ms/slice at batch size 1, and peak throughput was 1.48 ms/slice at batch size 512. In the separate 97-patient agreement cohort, the expert-adjudicated score was identical to the automated score for 967 of 1164 observations (83.1%) and differed for 197 (16.9%). In the modified-score subset, Pearson r was 0.918, mean absolute error was 3.07, and ICC(2,1) was 0.889 (patient-clustered 95% CI 0.828–0.923). The pooled ICC(2,1) was 0.988 (0.982–0.992), but this value was inflated because the 967 unchanged pairs were identical by construction. Sequential end-to-end processing, measured in seven study patients, took a mean of 22.5 s per patient (median 24.0 s, range 17.3–24.9 s); localisation accounted for 88.1% of this time. **Conclusions:** The framework combined lung segmentation, anatomically standardised sampling, and automated fibrosis scoring. The radial score showed preliminary analytical concordance under non-blind expert adjudication. The fibrosis detector remains a proof-of-concept implementation based on 8-bit windowed images and has not been compared with independently drawn pixel-level fibrosis masks. The radial partition is an exploratory scoring convention and was not compared with alternative partitions or validated against clinical outcomes. Larger external studies using native Hounsfield-unit data and independent blinded readers are required.

## 1. Introduction

Idiopathic pulmonary fibrosis (IPF) is a chronic, progressive interstitial lung disease [1] characterised by irreversible fibrosis of the lung parenchyma and a poor prognosis. Disease monitoring in clinical practice relies primarily on pulmonary function tests (PFTs), particularly forced vital capacity (FVC), and diffusing capacity for carbon monoxide (DLCO), as well as high-resolution computed tomography (HRCT) [2]. While declines in FVC are well-established predictors of mortality and are widely utilised as endpoints in clinical trials [3,4,5], PFTs and imaging assessments provide complementary insights by reflecting functional impairment and structural lung abnormalities, respectively.

HRCT enables the direct visualisation of fibrotic changes and has attracted increasing interest as a source of objective imaging biomarkers in IPF [6]. Traditional visual scoring systems, although clinically intuitive, often suffer from substantial inter-observer variability and limited reproducibility. Interobserver agreement is only moderate even for the categorical ATS/ERS/JRS/ALAT assessment of a UIP pattern [7], which is a coarser judgement than the extent grading a quantitative method has to reproduce. To address these limitations, several computer-assisted and automated approaches have been proposed to quantify the extent of fibrosis on CT scans. Early studies utilised threshold-based or histogram-derived metrics to estimate fibrotic burden, while more recent work has incorporated advanced machine learning and deep learning techniques [7,8,9,10] to segment lung parenchyma and identify fibrotic patterns. These approaches have demonstrated significant prognostic value and strong correlations with pulmonary function, underscoring the clinical potential of CT-based quantification.

Despite these technological advances, most existing methods implicitly assume adequate anatomical correspondence between serial CT examinations [11,12]. In clinical practice, longitudinal chest CT scans are often acquired at different breath-hold depths, producing non-rigid deformation of the lung parenchyma. Even after correction of a global axial (z-axis) offset, the tissue in nominally corresponding slices may undergo spatially heterogeneous displacement and local shape change. Rigid or translational alignment can compensate for global shifts but cannot restore intra-slice tissue correspondence. Deformable image registration can account for some non-rigid deformation, but it is computationally demanding and may introduce interpolation artefacts that bias quantification [11,12]. We therefore used predefined anatomical landmarks to standardise slice selection across examinations. This strategy promotes consistent anatomical sampling but does not establish voxel- or tissue-level correspondence.

The purpose of this study was therefore to develop and evaluate an automated CT-based framework for pulmonary fibrosis quantification using a standardised six-level anatomical protocol derived from the Taiwan ILD Semi-Quantitative Imaging Assessment Consensus [13]. By integrating YOLO11n-seg for lung segmentation, we aimed to generate standardised and deterministic per-level, per-lung fibrosis scores. We further examined analytical concordance between the automated radial score and a non-blind expert-adjudicated reference across 1164 observations from the final agreement cohort of 97 patients (84 IPF and 13 other ILD). This adjudication was not independent clinical validation.

This work makes four contributions. First, it implements the six-level Taiwan ILD consensus protocol in software and reports the internal accuracy of automated landmark localisation. Second, it combines that protocol with a lightweight lung-segmentation backbone and reports directly measured workflow timing rather than treating peak network throughput as end-to-end performance. Third, it reports two per-level, per-lung outputs: an area-weighted Fibrosis Index that is mathematically independent of the angular partition and an exploratory region-resolved radial score that retains angular distribution. Alternative partition schemes and associations with clinical outcomes and events were not evaluated. Fourth, it characterises concordance with a non-blinded expert-adjudicated reference and reports the modified-score subset separately from unchanged pairs that inflate pooled estimates. The study does not establish external clinical validity: the fibrosis detector is a proof of concept operating on 8-bit windowed images, no independent pixel-level fibrosis reference was available, and the physician assessment was a non-blinded adjudication rather than an independent reading.

## 2. Materials and Methods

### 2.1. Study Population

Institutional approval and waiver of informed consent: The study was conducted in accordance with the Declaration of Helsinki and approved by Research Ethics Committee I, China Medical University & Hospital, Taichung, Taiwan (approval code CMUH110-REC1-200; approval date 2 November 2021). The requirement for written informed consent was waived because of the retrospective design and the use of anonymised data.

**Figure 1:**
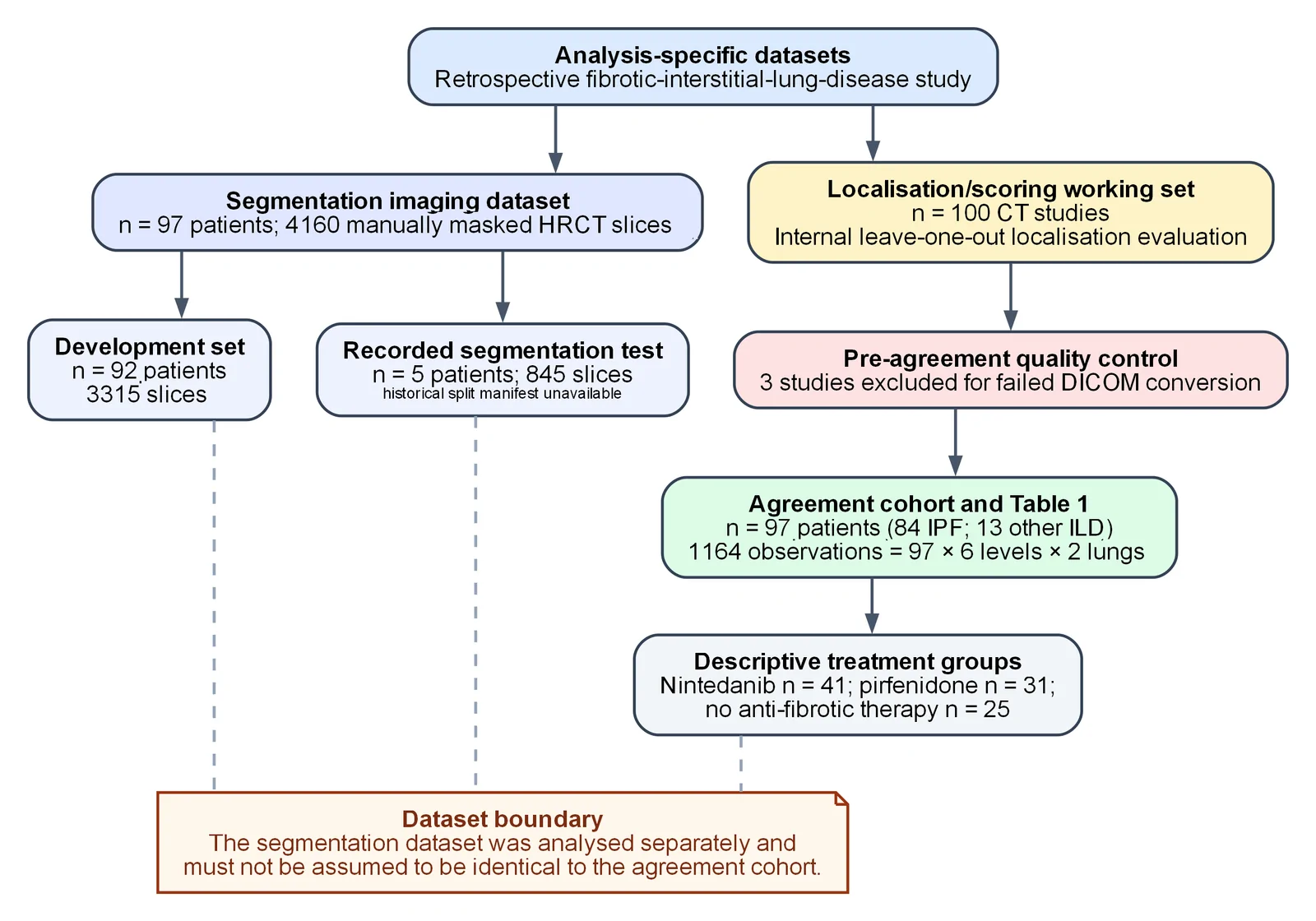
Analysis-specific datasets. Segmentation used 4160 slices from 97 patients (92 development and 5 internal-holdout patients from the same medical centre). A separate 100-study localisation/scoring set underwent internal leave-one-out localisation validation; 3 DICOM-conversion failures were excluded, leaving the 97-patient agreement cohort (84 IPF, 13 other ILD; 1164 observations) described in Table 1. In the diagram, n denotes the number of patients or CT studies specified in each box.

Patient cohort and analysis-specific datasets: The source clinical registry screened consecutive patients evaluated for fibrotic ILD between June 2017 and October 2021 with at least six months of follow-up. The segmentation dataset contained 4160 manually annotated HRCT slices from 97 patients, partitioned into 92 development patients and 5 internal-holdout patients from the same medical centre. Separately, a 100-study localisation/scoring set was evaluated by leave-one-out cross-validation; three studies were subsequently excluded from agreement analysis because DICOM conversion failed quality control, leaving 97 patients (84 IPF and 13 other ILD) and 1164 observations. Table 1 describes this final agreement cohort. The two analysis datasets were not assumed to contain identical patients. Imaging and clinical data were retrieved between January and October 2022 by investigators with access to identifiable information; subsequent analyses used anonymised data. Figure 1 summarises the analysis-specific datasets and their non-identical boundaries.

Clinical characteristics: Baseline clinical records for all 97 agreement-cohort patients were available for diagnosis, treatment group, sex, smoking status, recorded comorbidities, and HRCT pattern. Anthropometric and pulmonary function variables were analysed using available cases, with denominators reported below Table 1. Treatment status was descriptive only: 41 patients received nintedanib, 31 received pirfenidone, and 25 received no anti-fibrotic therapy.

### 2.2. Data Sets and Partitioning

Internal segmentation holdout: Five patients, each with at least two HRCT examinations, were selected and withheld before segmentation model development. Their 845 slices were used once for the final internal holdout evaluation. These scans came from the same medical centre as the development data and therefore do not constitute an external or independent validation cohort. Localisation was evaluated separately in the 100-study localisation/scoring set.

Segmentation training/validation set: The remaining 92 patients and 3315 slices constituted the training/validation pool for development of the segmentation and quantification algorithms. This segmentation dataset was analysed separately from the localisation/scoring set and final agreement cohort; patient identity across the analysis-specific datasets was not assumed.

### 2.3. CT Acquisition

All scans were acquired on multi-detector CT systems during a full-inspiratory breath-hold with the patient supine and with full thoracic coverage. Direct review was possible for 16 original DICOM series from seven study patients: in-plane pixel size ranged from 0.53 to 0.82 mm (median 0.66 mm), 97.0% of 1103 inter-slice gaps were 2.5 mm, and each series contained 59–74 axial 512 × 512 images spanning 158–188 mm. The exported archives did not retain a verifiable nominal section-thickness field; section thickness is therefore not asserted. Physician-annotated lung masks for all 4160 segmentation dataset slices served as the reference for the reported lung-segmentation metrics.

### 2.4. Segmentation Networks

Manual lung masks used for training were annotated by experienced thoracic radiologists and reviewed for consistency.

Five segmentation architectures from three model families were benchmarked under the reported preprocessing, augmentation, optimisation, and intersection-over-union loss settings:

1. Mask R-CNN [14] with a ResNet-101–FPN backbone.
2. U-Net derivatives: U-Net [15], BT-U-Net [16], Ladder-Net [17].
3. YOLO11n-Seg [18]. Several YOLO11-seg scales (*n* to x) were examined during preliminary development. Their mean cross-validation mIoU values were comparable, and the nano variant was selected because it had the lowest measured computational burden and fastest inference among the examined scales. This engineering comparison did not establish clinical workflow performance or equivalent external generalisation.

**Figure 2:**
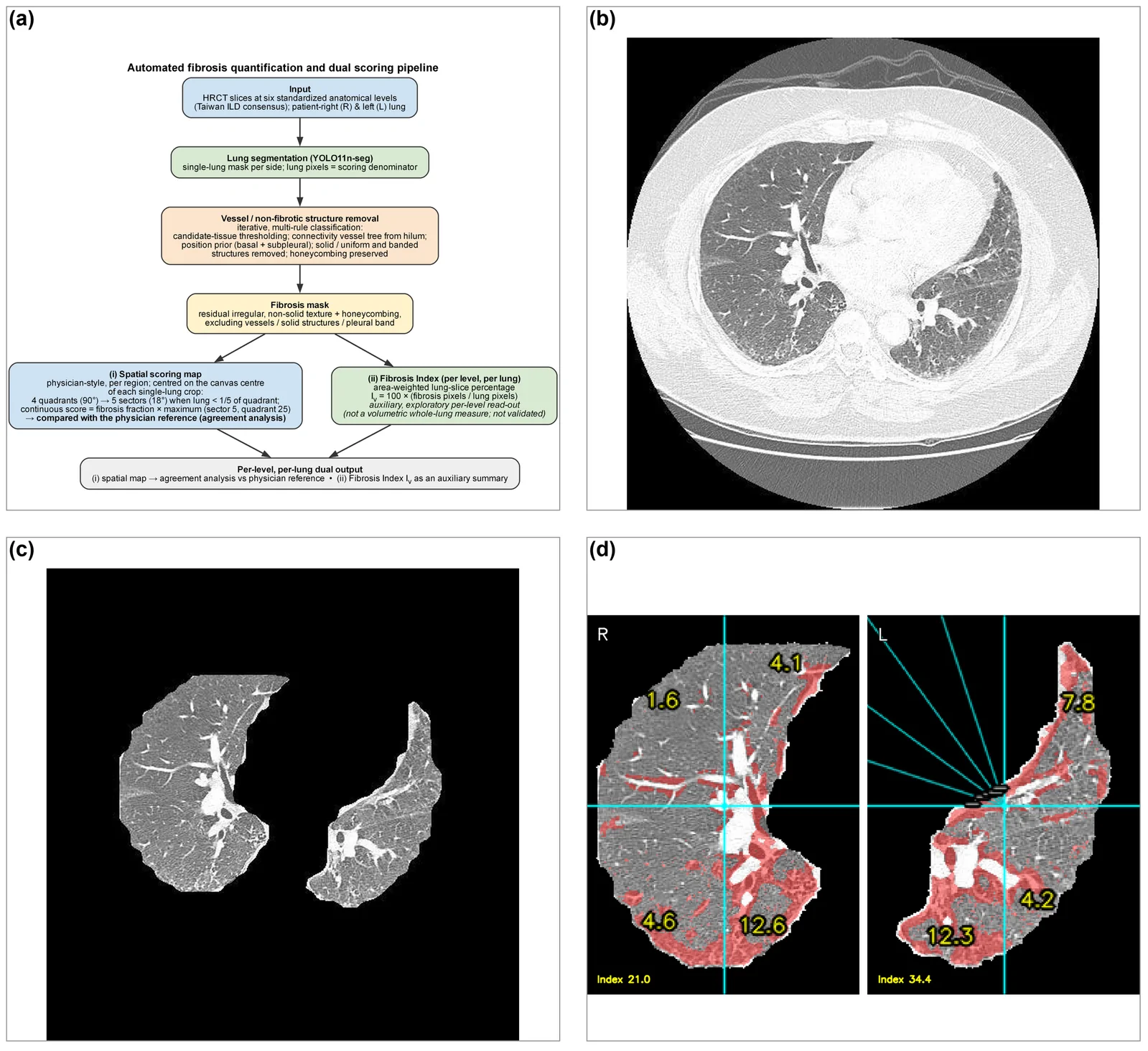
Automated framework and same-slice representative processing chain. In panel (**a**), ‘basal’ is the legacy name of implementation variable P_basal; as defined in Appendix A.7, it represents the vertical image position within the crop rather than a true three-dimensional cranio-caudal coordinate. (**a**) Workflow from HRCT input and lung segmentation to structure removal, fibrosis masking, spatial scoring, and the auxiliary per-level, per-lung area-weighted Fibrosis Index $I_{v}$. The subscript v is a fixed index label and does not denote volume (Equation (3)). (**b**) Representative 8-bit windowed HRCT at one anatomical level. (**c**) Bilateral lung mask from the same slice; lung pixels define the per-lung denominators. (**d**) Patient-right (R) and patient-left (L) outputs generated from the same input and masks as (**b**,**c**). Red indicates retained fibrosis, cyan the regional partition, and yellow the spatial scores; the displayed “Index” is $I_{v}$. Intermediate removal stages are shown in Figure 3. This panel illustrates the processing chain and is not independent validation.

Training and inference were performed on an NVIDIA RTX A6000 GPU (NVIDIA Corporation, Santa Clara, CA, USA; 48 GB). Mean IoU and model-only inference time for 512 × 512 inputs were recorded. YOLO11n-seg was fastest, U-Net derivatives intermediate, and Mask R-CNN slowest in the reported engineering benchmark. These latency and throughput values exclude image I/O, anatomical localisation, fibrosis post-processing, and report generation. The complete segmentation-to-scoring workflow and representative same-slice processing outputs are summarised in Figure 2.

**Figure 3:**
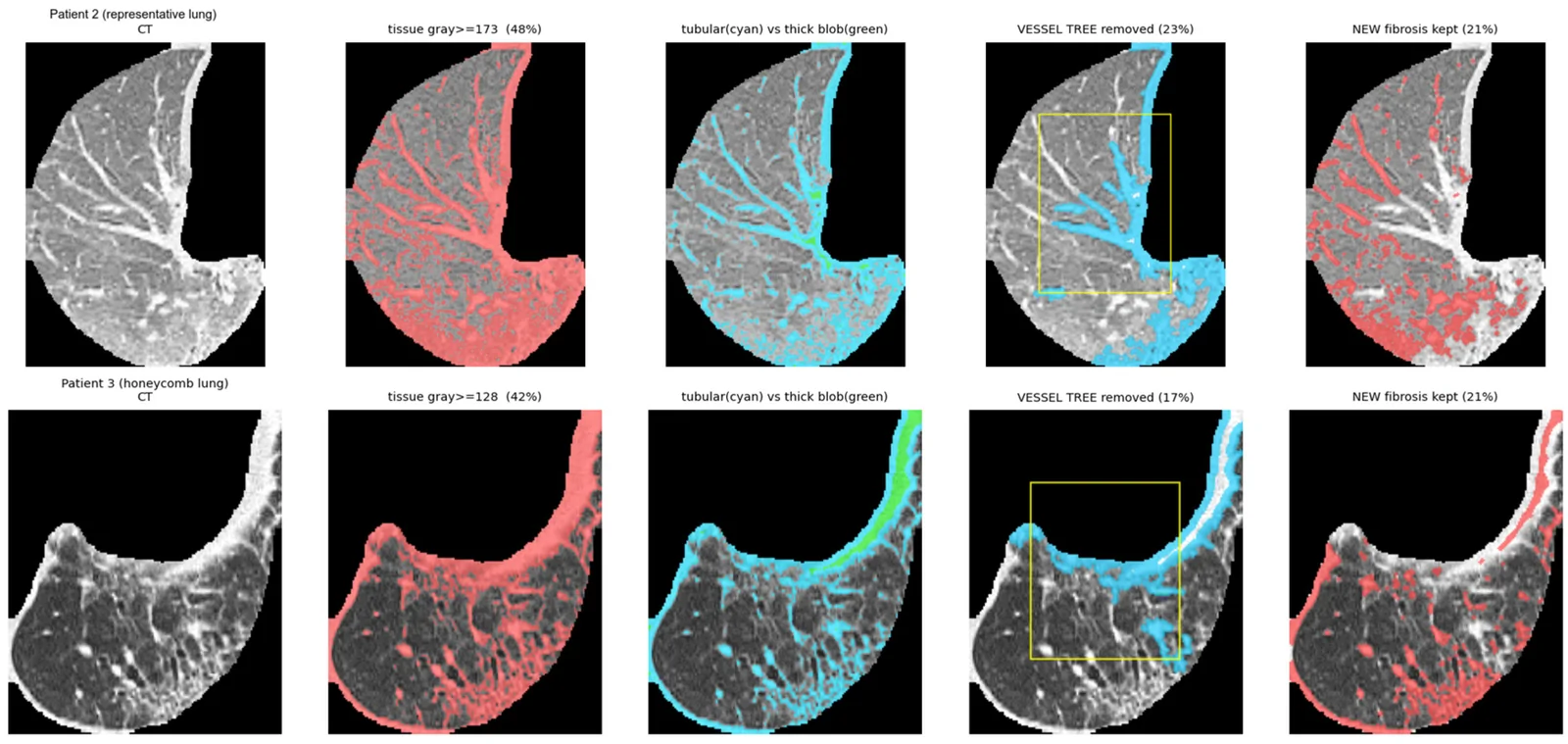
Fibrosis detection by vessel- and structure-removal in two representative lungs (Patient 2, top; honeycomb-predominant Patient 3, bottom): from left to right, the input CT; candidate tissue above the grey-level threshold; the multi-scale tubular (cyan) versus blob/solid (green) response; the connectivity-based vessel tree removed (yellow box = hilar seed region); and the residual texture retained as fibrosis. In the panel labels, 173 and 128 are case-specific thresholds on the 8-bit grayscale intensity, and every percentage is the corresponding pixel count divided by the lung mask pixel count. The top row is not a normal lung reference. It illustrates residual signal retained by the current 8-bit pipeline, including incompletely removed peripheral vessels and other false-positive tissue. Native Hounsfield-unit input is required for further validation.

### 2.5. Three-Step Image Pre-Processing and Mask Extraction

Notation: Let j index scans; let $N_{j}^{slice}$ be the number of axial slices in scan j; let s index slices ($1\leq s\leq N_{j}^{slice}$); and let q∈{L,R} denote the patient-left or patient-right lung.

$G_{j,s}$: 8-bit grey-scale image (512 × 512) (Figure 2b).

$M_{j,s}^{L}$, $M_{j,s}^{R}$: binary masks of the left and right lungs are generated by the chosen network.

$M_{j,s}^{fib}$: binary fibrosis mask.

Step 1: Image conversion: All DICOM slices were windowed (width/level = 1500/−600 HU) and exported as 8-bit PNG images $G_{j,s}$. Under this window −1350 HU maps to 0 and +150 HU to 255.

Step 2: Lung masking (Figure 2c): The network probability map was thresholded to obtain $M_{j,s}^{L}$ and $M_{j,s}^{R}$.

Step 3: Preliminary fibrosis mask and spatial-scoring output (Figure 2d and Figure 3): Pixel intensity thresholding alone could not delineate fibrosis. On 8-bit windowed images the grey levels of fibrosis, intraparenchymal vessels, and normal high-attenuation parenchyma overlap, so a fixed intensity interval simultaneously discards genuine subpleural fibrosis and retains vessels. We therefore only used intensity to generate candidate tissue, followed by the structure-removal strategy detailed below. Figure 3 shows the intermediate removal stages. Figure 2d was generated from the same HRCT slice and lung masks shown in Figure 2b,c and displays the final retained fibrosis pixels (red), regional guides (cyan), spatial scores (yellow), and the auxiliary per-level Fibrosis Index.

Bright non-fibrotic structures include airway walls and the pulmonary vasculature, which commonly form continuous tubular, band-like, or compact components on axial images. We identified them by combining a multi-scale Hessian (Frangi-type) vesselness response, a connectivity-based vessel tree grown from the hilum, and component-level solidity descriptors, including local intensity uniformity, convexity, and circularity. Continuous dense bands, compact block-like regions, and uniformly solid components were removed as non-fibrotic structures. The classification therefore relied on spatial continuity and solidity in addition to intensity. Honeycombing, defined as clustered small dark cysts bounded by bright walls, was explicitly protected from removal.

Because vessels and fibrosis can have similar brightness on 8-bit windowed images, the algorithm also applies a soft positional prior. Within each axial single-lung crop, peripheral and lower-image regions are weighted more strongly as fibrosis candidates. This is a two-dimensional image-coordinate cue; it does not replace the cranio-caudal information supplied by the six anatomical levels. The prior resolves competing cues near the pleural margin, where vessels taper but subpleural fibrosis is also common. The agreement cohort included 13 patients with non-IPF ILD, but diagnosis-specific performance was not evaluated; applicability to non-UIP distributions remains uncertain.

These cues are not applied as a single feed-forward threshold but as an iterative, multi-rule procedure in which the candidate label of each region (normal, vessel, pleura, or fibrosis) is repeatedly cross-checked and revised. A final position-reasonableness check removes any fibrosis label whose location, shape (solid, blocky, or band-like) or connectivity is inconsistent with fibrotic tissue. The residual irregular, non-solid texture within the lung field is retained as the fibrosis estimate.

The resulting binary fibrosis mask was restricted to the corresponding lung mask, so pixels outside the lung could not enter quantification. The present implementation uses 8-bit images exported for visual review. Migration to native Hounsfield-unit data will require recalibration of intensity-domain thresholds, whereas percentile-based and geometric rules can be retained (Appendix A.12). Native-HU implementation is expected but has not yet been shown to improve separation of vessels, consolidation, and fibrotic tissue.

### 2.6. Standardised Six-Level Anatomical Scoring Protocol

Serial chest CT examinations in patients with IPF are often acquired at different inspiratory volumes. The resulting parenchymal deformation is non-rigid and spatially heterogeneous. Even when two examinations are aligned along the z-axis, local tissue displacement, rotation, and shape change remain within the selected axial planes. Lung-area matching or a simple z-offset can correct a global cranio-caudal shift but cannot ensure that the same tissue is being compared. We therefore anchored sampling to predefined anatomical landmarks. This is a pragmatic standardisation strategy, not a registration method, and it does not establish longitudinal repeatability or tissue-level correspondence.

Following the Taiwan Interstitial Lung Disease Semi-Quantitative Imaging Assessment Consensus [13], we adopted a standardised six-level anatomical protocol: (1) the superior border of the aortic arch, (2) 1 cm below the carina, (3) the pulmonary venous confluence, (4) halfway between levels 3 and 5, (5) 1 cm above the right hemidiaphragm, and (6) 2 cm below the right hemidiaphragm. The lungs were scored independently at each level. An experienced thoracic radiologist visually estimated the percentage of lung parenchyma exhibiting honeycombing, reticulation, traction bronchiectasis, and ground-glass opacity with traction bronchiectasis, using increments of at least 5 percentage points. The same levels were located automatically; the radial quadrant/sector score was the quantity adjudicated against the expert reference, while the Fibrosis Index was retained as an auxiliary per-level, per-lung read-out rather than a volumetric whole-lung measure. Each patient contributed 12 paired radial-score observations (6 levels × 2 lungs).

Automated localisation of the six anatomical levels used a hybrid two-stage algorithm. First, eight anchor positions were extracted per CT volume: four classical anchors from lung-mask geometry and grayscale morphology (the superior and inferior lung extents, carinal bifurcation, and right hemidiaphragm) and four additional anchors from a complementary YOLOv12 [19] six-class detector trained to recognise upper-mediastinal, carinal-transition, mid-thoracic, and lower-mediastinal landmark regions, using the maximum-confidence and last-firing slices of the relevant classes. Second, six independent ordinary-least-squares models fitted on a 100-study calibration set predicted the six slice indices from the eight anchors. An experienced thoracic radiologist selected the reference slices according to the Taiwan ILD consensus. Predicted indices were rounded, bounded to the available slice range, and post-processed to enforce $L_{1}<L_{2}<\ldots <L_{6}$ before scoring.

Localisation accuracy was assessed internally on the 100-study calibration set by leave-one-out cross-validation. Mean absolute errors were 1.7, 2.6, 3.8, 2.9, 2.9, and 3.0 slices for $L_{1}–L_{6}$, respectively, and median absolute error was below three slices at every level. The proportion within five slices of the radiologist reference ranged from 71% at $L_{3}$ to 95% at $L_{1}$ (mean 84%), supporting use of the algorithm as the upstream localisation step within this dataset (Table 2); external validation remains necessary.

**Table 2:** Per-level localisation accuracy of the automated six-level algorithm (leave-one-out cross-validation, 100 CT studies). MAE: mean absolute error; *n*: number of CT studies.

Anatomical Level	MAE (Slices)	Median (Slices)	Within 3 Slices, *n* (%)	Within 5 Slices, *n* (%)
L1 (aortic arch)	1.7	1.3	84 (84.0%)	95 (95.0%)
L2 (carina + 1 cm)	2.6	2.2	66 (66.0%)	90 (90.0%)
L3 (pulmonary venous confluence)	3.8	2.9	54 (54.0%)	71 (71.0%)
L4 (midpoint)	2.9	2.3	61 (61.0%)	82 (82.0%)
L5 (right hemidiaphragm − 1 cm)	2.9	2.0	68 (68.0%)	83 (83.0%)
L6 (right hemidiaphragm + 2 cm)	3.0	2.4	60 (60.0%)	85 (85.0%)

### 2.7. Quantification of Fibrotic Burden

Per-level and per-slice quantities: At each anatomical level l∈{1,…,6}, let s(j,l) denote the selected axial slice in scan j. For any binary mask M, |M| denotes its number of pixels. Let $A_{px,j,s}$ denote the in-plane area represented by one pixel at slice s of scan j (for example, mm^2^ per pixel, obtained from DICOM Pixel Spacing). Then:

(1) $$A_{j,s}^{q}=A_{px,j,s}\left|M_{j,s}^{q}\right|,\;\;q\in \{L,R\}$$

(2) $$n_{j,l}^{fib,q}=\left|M_{j,s(j,l)}^{fib}\cap M_{j,s(j,l)}^{q}\right|,\;n_{j,l}^{lung,q}=\left|M_{j,s(j,l)}^{q}\right|,\;q\in \{L,R\}$$

Per-level per-lung Fibrosis Index (percentage; 0–100):

(3) $$I_{v,j,l}^{q}=100\;\frac{n_{j,l}^{fib,q}}{n_{j,l}^{lung,q}},\;\;q\in \{L,R\}$$

Combined left-right Fibrosis Index (percentage; 0–100; unnumbered definition):

$$I_{v,j,l}=100\;\frac{n_{j,l}^{fib,L}+n_{j,l}^{fib,R}}{n_{j,l}^{lung,L}+n_{j,l}^{lung,R}}$$

The per-level side-to-side difference $\Delta I_{v,j,l}=I_{v,j,l}^{R}-I_{v,j,l}^{L}$ is expressed in percentage points and permits assessment of asymmetrical disease at each anatomical level.

In addition to the area-weighted index, the framework produces an exploratory spatial map patterned after the regional layout of the Taiwan ILD consensus [13]. This correspondence defines the display convention but does not constitute evidence that the radial partition is superior to rectangular or other partition schemes. However, the reference in this study was recorded once per lung at each selected level, not as independently assessed region-level values. Within each square single-lung crop, horizontal and vertical lines through the fixed canvas centre define four 90° quadrants. A quadrant is scored as a whole when lung occupies at least one fifth of its area; otherwise, it is subdivided into five 18° sectors, and only sectors exceeding the same occupancy threshold are evaluated. Each evaluated region receives a continuous lung-normalised score weighted by angular span—up to 25 for a quadrant and 5 for a sector—so the per-lung ceiling is 100. For a quadrant or sector R, $N_{R}$ is its total pixel count, $n_{R}^{lung}$ and $n_{R}^{fib}$ are its lung- and fibrosis-pixel counts, and $w_{R}$ is its angular weight:

(4) $$\phi_{R}=\frac{n_{R}^{lung}}{N_{R}}$$

(5) $$\sigma_{R}=\frac{n_{R}^{fib}}{n_{R}^{lung}}\;w_{R}=p_{R}w_{R}$$

(6) $$S_{j,l}^{q}=\sum_{R\in E_{j,l}^{q}}\sigma_{R}\leq 100$$

In Equations (4)–(6), $\phi_{R}=\frac{n_{R}^{lung}}{N_{R}}$ is the lung-occupancy fraction and $p_{R}=\frac{n_{R}^{fib}}{n_{R}^{lung}}$ is the fibrosis fraction among lung pixels; $\sigma_{R}=p_{R}w_{R}$ is the weighted regional score. The evaluation set $E_{j,l}^{q}$ contains a quadrant R when $\phi_{R}\geq \frac{1}{5}$. If that quadrant fails the threshold and is subdivided, a sector R is included only when $\phi_{R}>\frac{1}{5}$. $S_{j,l}^{q}$ is the sum over the applicable evaluation set for one lung q∈{L,R}.

The one-fifth occupancy threshold is a safeguard against unreliable estimates. Each regional fibrosis fraction $p_{R}=\frac{n_{R}^{fib}}{n_{R}^{lung}}$ has precision governed by its denominator; under a binomial approximation, its variance is of order $\frac{p_{R}(1-p_{R})}{n_{R}^{lung}}$. Sparsely filled regions tend to lie at lung boundaries and crop corners, where partial-volume averaging and residual segmentation boundary error are largest, so a few pixels can shift the estimate disproportionately. Regions below the applicable quadrant or sector threshold are therefore reported as not evaluated rather than assigned false precision.

The four-quadrant partition with conditional 18° subdivision was an implementation-defined display and scoring convention. It was selected to provide a deterministic regional decomposition and to mirror a familiar regional layout; this study did not establish that it is scientifically or clinically superior to rectangular or other partition schemes. We did not compare scores from alternative partitions or test their associations with clinically important outcomes or events. Such validation would require prespecified competing partitions, independent regional references and/or clinically important outcomes, and formal statistical comparison. Accordingly, the radial partition is treated here as exploratory.

Tying each region’s maximum attainable value to its angular coverage places the adaptive partition on a single interpretable scale. Because the partition adapts to local lung-fill, the number and angular width of the regions actually evaluated for a given lung are data-dependent and vary between lungs, slices, and patients, so a score formed by naively summing per-region values would depend partly on how the lung happened to be partitioned rather than on disease burden alone. To prevent this, each region is allotted a budget equal to its share of the 360° field, a 90° quadrant receiving $\frac{90}{360}$ of the per-lung budget of 100 (that is, 25) and an 18° sector receiving $\frac{18}{360}$ (that is, 5), so that a fully involved single lung has a fixed ceiling of 100. This allocation is conserved under refinement since a quadrant and the five sectors into which it may be split carry the same aggregate budget (25=5×5); considered purely as a reallocation of weight, the adaptive split therefore neither inflates nor deflates the per-lung ceiling. The realised total of 100 is attained only when every region clears the occupancy threshold and is fully involved, whereas sub-threshold regions forfeit their budget and lower the attained total accordingly, which anchors the coarse quadrant-level number and the finer sector-level decomposition on one common 0–100 scale that is comparable across lungs and across the six anatomical levels.

Each evaluated region receives a continuous score derived from its lung-normalised fibrosis fraction and angular weight. This deterministic implementation avoids ordinal cut-points, but it is a design choice rather than a validated clinical weighting scheme. Continuous radial scoring was not compared with rectangular or other partitions, and its association with clinical outcomes or events was not evaluated.

The two read-outs are reported together to document distinct outputs of the implementation; the study did not test whether either partitioned representation is clinically advantageous. The area-weighted Fibrosis Index of Equation (3) counts every lung pixel at one selected anatomical level, giving a spatially unweighted per-level, per-lung scalar that is free of the regional weighting and sub-threshold drop-out affecting the angular map; it is auxiliary and was not validated against the physician scores. The spatial map S is the exploratory region-resolved map from which the per-lung score was derived and adjudicated; neither its individual regions nor its weighting scheme was independently validated. The two read-outs are not generally equal: the map omits sub-threshold regions and uses angular weights, whereas the Index integrates the complete per-level lung mask using lung-pixel-area weighting. Their difference is therefore an expected design consequence rather than an implementation inconsistency.

Finally, radial division is centred on the fixed canvas centre of each square single-lung crop, $\left(\frac{w-1}{2},\frac{h-1}{2}\right)$, which matches the deterministic implementation of the horizontal/vertical cross and radial fan. Here, w and h denote the crop width and height in pixels. This deterministic origin is applied identically across slices and patients. The two lungs are partitioned separately because they are anatomically distinct fields with different shapes, centres, and disease distributions; a merged partition could place bins across the mediastinum and obscure left–right asymmetry. The two lungs of a patient were not treated as statistically independent; within-subject correlation was handled in the repeated-measures analysis.

In current practice, interval change is judged by comparing serial examinations, and estimating whether the affected proportion has increased. The present method instead produces fixed-scale per-level, per-lung values from fibrosis- and lung-pixel counts. In this feasibility study, a single experienced thoracic radiologist examined each displayed overlay and recorded one expert score per lung while the automated candidate value was visible. The resulting reference was therefore a non-blinded expert-adjudicated measurement, not independent pixel-level ground truth and not evidence of cross-institutional comparability.

Agreement evaluation: An experienced thoracic radiologist evaluated 600 anatomical-level displays from the 100-study localisation/scoring set. Each display paired the input CT with the fibrosis overlay, radial partition, and regional scores for both lungs. Three studies were excluded after DICOM-conversion quality control, leaving 582 anatomical levels from 97 patients (84 IPF and 13 other ILD), or 1164 per-level, per-lung radial scores. For each lung, the radiologist recorded one expert score after viewing the overlay and automated candidate value. Identical automated and expert values formed the unchanged subset; observations in which the expert value differed formed the modified-score subset. Because the overlay and candidate value were visible, the reference was a non-blinded adjudication rather than an independent reading. The reference comprised one value per lung plus an anatomical-level narrative finding; it was not an independently drawn fibrosis mask.

The primary summary was the proportion of unchanged and modified scores, with a patient-clustered bootstrap 95% confidence interval for the unchanged proportion. Additional analyses included repeated-measures within-subject correlation; Pearson and Spearman correlations with patient-clustered bootstrap confidence intervals; ICC(2,1) for absolute agreement and ICC(3,1) for consistency; mean absolute error; root-mean-square error; mean signed bias; and descriptive Bland–Altman limits of agreement. ICCs were calculated for all observations, for the modified-score subset, and per patient. Error and agreement metrics were reported for both the complete set and the 197 modified observations because the 967 unchanged pairs were identical by construction and inflated pooled estimates.

### 2.8. Statistical Analysis

Statistical analyses were descriptive and exploratory. Continuous baseline variables are presented as median (interquartile range) and compared among treatment groups by Kruskal–Wallis tests; categorical variables are counts (%) and compared by Pearson $\chi^{2}$ or Fisher–Freeman–Halton exact tests as appropriate. Agreement confidence intervals used patient-cluster resampling. Tests were two-sided with p<0.05. Baseline analyses were performed with MedCalc version 18.10 (MedCalc Software Ltd., Ostend, Belgium); agreement and bootstrap calculations used Python version 3.11.8 (Python Software Foundation, Beaverton, OR, USA).

Nomenclature Summary:

$G_{j,s}$: 8-bit grey-scale CT slice.

$M_{j,s}^{L}$, $M_{j,s}^{R}$: binary masks of the patient-left and patient-right lungs.

$M_{j,s}^{fib}$: binary fibrosis mask.

$A_{j,s}^{q}$: physical lung area in one slice; $A_{px,j,s}$ is in-plane area per pixel and |⋅| denotes mask cardinality (Equation (1)).

$n_{j,l}^{fib,q},\;n_{j,l}^{lung,q}$: fibrosis- and lung-pixel counts at anatomical level l (Equation (2)).

$I_{v,j,l}^{L},\;I_{v,j,l}^{R}$: per-level, per-lung Fibrosis Index percentages on a 0–100 scale (Equation (3)); the fixed label v distinguishes this Index from spatial score S and is not a running index.

Indices and operators: j indexes scans; $N_{j}^{slice}$ is the number of axial slices in scan j; s indexes slices; l indexes the six anatomical levels; q∈{L,R} indexes patient side; and s(j,l) is the selected slice at level l.

Regional symbols: R is a quadrant or adaptively subdivided sector; $N_{R}$ is its total pixel count; $\phi_{R}=\frac{n_{R}^{lung}}{N_{R}}$ defines lung occupancy; $p_{R}=\frac{n_{R}^{fib}}{n_{R}^{lung}}$ defines the fibrosis fraction among lung pixels; $w_{R}$ is the angular weight; $\sigma_{R}=p_{R}w_{R}$ defines the weighted regional score; $E_{j,l}^{q}$ is the set of evaluated regions; and $S_{j,l}^{q}$ is the per-lung spatial score.

The analyses used distinct datasets. The segmentation dataset comprised 4160 HRCT slices: 3315 development slices from 92 patients and a non-overlapping internal holdout of 845 slices from five patients. Separately, the six-level localisation/scoring set contained 100 studies. After three DICOM-conversion quality-control exclusions, the final agreement cohort contained 97 patients (84 IPF and 13 other ILD), each contributing 12 per-level, per-lung scores, for 1164 observations. Table 1 reports this agreement cohort; the segmentation dataset was not assumed to contain the same patients.

## 3. Results

### 3.1. Data Sets

The segmentation dataset comprised 4160 HRCT slices. This included 3315 development slices from 92 patients (median 33 slices per patient; IQR 26–40) for segmentation model training and validation and a non-overlapping internal holdout of 845 slices from 5 patients (median 153 slices per patient; IQR 129–220) for final evaluation. The five internal-holdout patients, each with at least two HRCT scans, were selected before segmentation model evaluation.

### 3.2. Key Findings from the Segmentation Benchmark

On an NVIDIA RTX A6000 GPU, YOLO11n-seg converged in approximately 35 min. Model-only latency was 73.8 ± 9.7 ms/slice at batch size 1, and peak throughput was 1.48 ms/slice at batch size 512 (Table 3). A separate sequential end-to-end benchmark using the original DICOM archives of seven study patients took a mean of 22.5 s per patient (median 24.0 s, range 17.3–24.9 s). The stage times were 0.086 s for header scanning (0.4%), 0.238 s for decoding (1.1%), 0.281 s for preprocessing (1.2%), 19.839 s for six-level localisation (88.1%), 0.490 s for segmentation of the six selected levels (2.2%), 1.492 s for fibrosis detection (6.6%), and 0.080 s for scoring (0.4%). Model loading and warm-up required an additional 14.8 s once per session and were excluded from per-patient timing. Batched localisation passes were measured at 3.48 s rather than 19.84 s; substituting those passes projects an end-to-end time of approximately 6.1 s per patient, although the fully integrated batched workflow was not timed directly. Table 4 shows that YOLO11n-seg had the smallest parameter count and lowest reported computational load among the compared models; these engineering measurements do not establish clinical workflow performance.

The 10-fold cross-validation result for the 92-patient development pool is summarised in Table 5.

Seg mentation Accuracy and Model Selection

We selected YOLO11n-seg as the primary segmentation backbone. Preliminary 10-fold cross-validation produced similar mean mIoU values across the examined YOLO11-seg scales (n to x); the nano variant had the lowest measured computational burden and fastest model-only inference in this comparison.

The model achieved an in-sample mIoU of 0.95 on the full training set (Table 4) and a 10-fold cross-validation mIoU of 0.9402 ± 0.0044 on the 3315-slice development set from 92 patients (Table 5), using physician-annotated lung masks as the reference. Table 4 also reports lower model-only latency and peak-throughput times for YOLO11n-seg than for the comparator architectures. These values describe the engineering benchmark and should not be interpreted as measured end-to-end clinical performance.

Evaluation Strategy: 10-Fold Cross-Validation and Internal Holdout Testing

We used a two-stage internal evaluation. First, 10-fold cross-validation on the 3315-slice development set was used for model selection. Second, the selected model was evaluated once on the 845-slice internal holdout from five patients, which had not been used for training, cross-validation, or hyperparameter selection. The holdout mIoU was 0.926 ± 0.017 (Table 6). Because the holdout contained only five patients, this estimate is preliminary and does not establish external or real-world generalisation.

**Table 6:** Segmentation performance on the internal holdout (five patients). mIoU: mean intersection over union; SD: standard deviation; ms: milliseconds.

Patient ID	Number of Slices	Total Lung mIoU	Left Lung mIoU	Right Lung mIoU	Inference Time (ms/Slice)
Patient 1	129	0.938	0.938	0.938	56.9
Patient 2	221	0.935	0.941	0.929	74.3
Patient 3	220	0.942	0.948	0.936	80.1
Patient 4	153	0.903	0.909	0.898	79.4
Patient 5	122	0.914	0.918	0.911	78.1
M ean ± SD	Total: 845	0.926 ± 0.017	0.931 ± 0.017	0.922 ± 0.017	73.8 ± 9.7

#### 3.2.1. Data Partitioning

Internal holdout. Scans from 5 of the 97 patients in the segmentation dataset (≈5%) were set aside once, before model development, and were not accessed during segmentation training or hyperparameter tuning.

Training/validation pool. The remaining 92 segmentation dataset patients were retained for all model selection procedures.

#### 3.2.2. Ten-Fold Cross-Validation on the Training Pool

The 92 development patients were randomly split into ten mutually exclusive folds. In each iteration, the model was trained on nine folds and evaluated on the remaining fold. This produced out-of-fold predictions and ten fold-level scores used for hyperparameter optimisation, early stopping, and model comparison. These values are internal development set estimates; the non-overlapping five-patient internal segmentation holdout remained untouched during these procedures, reducing leakage between model development and final testing. Because it came from the same medical centre, it was not treated as evidence of external generalisability.

#### 3.2.3. Statistical Assessment

Effect estimates and 95% confidence intervals are reported where the available analysis supports them.

#### 3.2.4. Final Model and Internal Holdout Evaluation

After selecting the hyper-parameter configuration that achieved the best average CV performance, the model was retrained from scratch on the full pool of 92 patients and then evaluated once on the 5-patient internal holdout.

Restricting cross-validation to the development data and evaluating the selected final model once on the five-patient holdout provided an internal holdout estimate and reduced the risk of optimistic reporting. The small holdout nevertheless limits precision and does not establish external generalisation.

**Figure 4:**
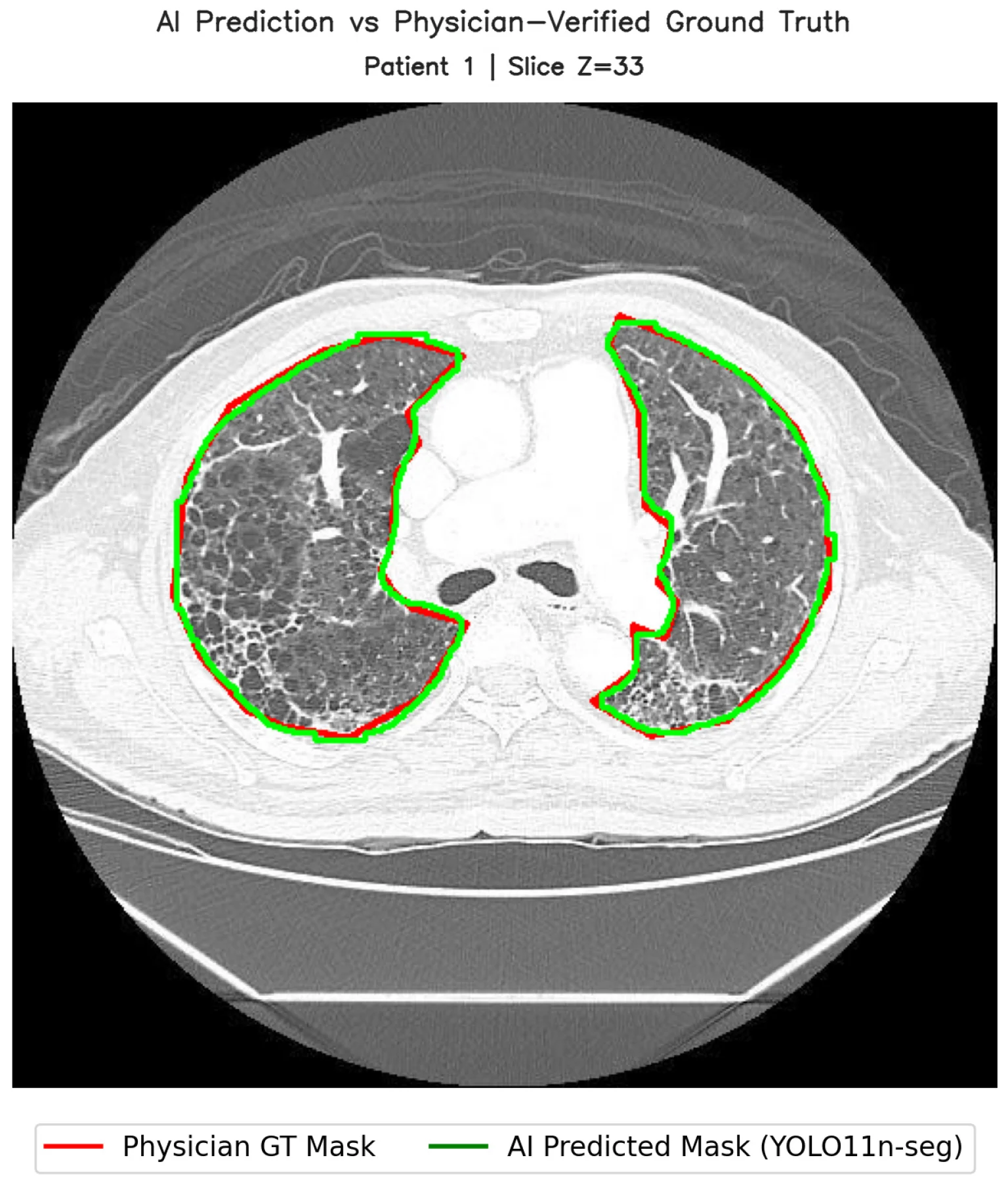
Qualitative comparison between an AI-predicted lung mask and physician-verified ground truth. The red contour is the physician ground-truth (GT) mask, and the green contour is the YOLO11n-seg prediction. The image label Z=33 denotes axial slice index 33 for Patient 1. This single example illustrates model behaviour and is not itself a quantitative performance measure.

Figure 4 provides a representative qualitative comparison between a YOLO11n-seg lung mask and the physician-annotated reference on axial slice 33 from Patient 1. The close contour overlap in this example illustrates model behaviour on one slice but is not an additional quantitative performance estimate.

### 3.3. Agreement Between the AI-Derived and Expert-Adjudicated Semi-Quantitative Fibrosis Scores

The adjudication records documented consideration of both the displayed delineation and the numeric score. Each of the 197 modified observations included an anatomical-level narrative finding that identified a target and usually a discrepancy mechanism, such as a retained vessel, missed parenchymal abnormality, contour-only detection, or marking outside the lung field. An additional eight observations included a recorded finding without a numeric difference. Findings were stored once per anatomical level, whereas scores were stored per lung; among 94 findings that named one lung, 91 (96.8%) coincided with modification of that lung’s score and none coincided with modification of the contralateral score alone. These records support internal consistency of the adjudication process but do not provide independent pixel-level validation. The reference retained one value per lung, so opposing regional errors could cancel within a lung and smooth boundary bias could be missed. Dice or IoU against an independently drawn fibrosis mask was therefore not computable and is not reported.

**Figure 5:**
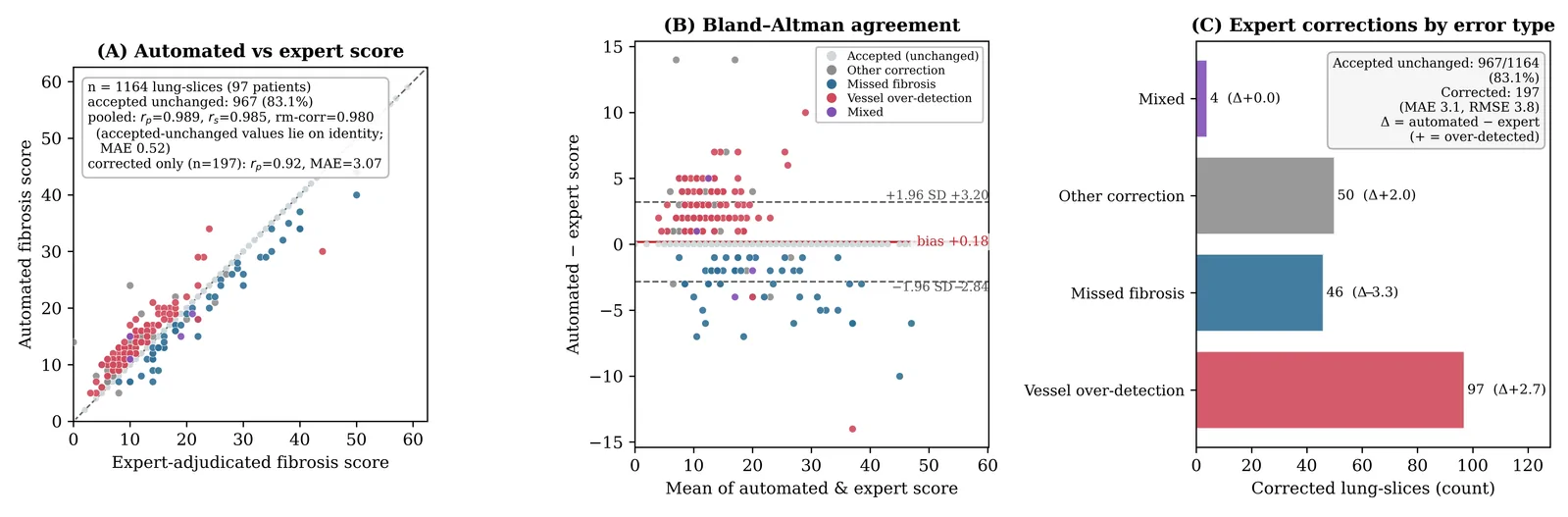
Agreement between the automated per-lung semi-quantitative fibrosis score and the expert-adjudicated reference (1164 lung-slice observations from 97 patients: 97 × 6 levels × 2 lungs). (**A**) Automated versus expert score with the line of identity; points are coloured by adjudication status. light grey = unchanged after adjudication; grey = other correction; blue = missed fibrosis; red = vessel over-detection; and purple = mixed. (**B**) Bland–Altman plot of the automated-minus-expert difference against their mean, with mean bias (solid line) and ±1.96 standard deviation limits of agreement (dashed lines). (**C**) Modified observations categorised by discrepancy type. In the annotations, $r_{p}$, $r_{s}$, and $r_{m-corr}$ denote the Pearson, Spearman rank, and repeated-measures within-subject correlation coefficients, respectively; MAE is mean absolute error, RMSE is root mean square error, and SD is standard deviation. Δ = automated − expert (positive values indicate over-detection). Point colours are defined by the legend in (**B**).

Of the 1164 observations, 967 were unchanged after adjudication (83.1%; patient-clustered bootstrap 95% CI 79.5–86.5%) and 197 had modified expert scores (16.9%). In the modified-score subset, Pearson r was 0.918 (95% CI 0.862–0.953), Spearman r was 0.861 (95% CI 0.772–0.921), ICC(2,1) was 0.889 (95% CI 0.828–0.923), ICC(3,1) was 0.896, MAE was 3.07, RMSE was 3.77, mean bias was +1.09, and descriptive Bland–Altman limits were −6.01 to +8.18. In the pooled analysis, repeated-measures within-subject correlation was 0.980; Pearson r was 0.989, Spearman r was 0.985, ICC(2,1) and ICC(3,1) were both 0.988, MAE was 0.52, RMSE was 1.55, mean bias was +0.18, and limits were −2.84 to +3.20 (Figure 5A,B). For the 967 unchanged pairs, ICC was exactly 1.000 and MAE was 0 by construction. The modified-score subset therefore provides the more informative agreement estimates. Per-patient ICC(2,1) varied substantially (median 0.986, IQR 0.960–1.000, range 0.123–1.000). These analyses do not represent blinded inter-reader agreement.

**Figure 6:**
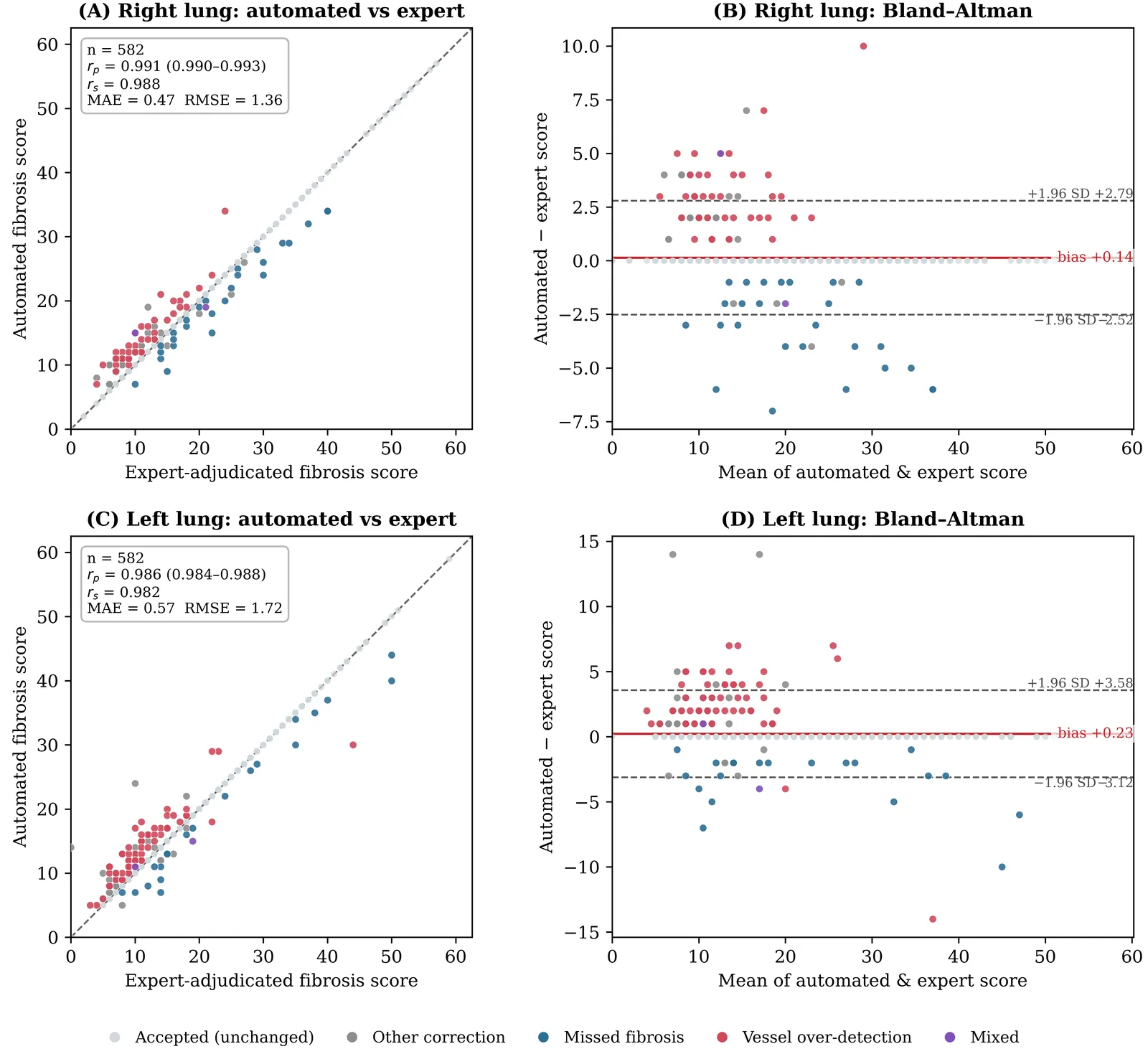
Right- and left-lung agreement analysed separately (582 lung-slice observations per side). (**A**,**B**) Right lung and (**C**,**D**) left lung, each shown as an automated-versus-expert scatter with line of identity and a Bland–Altman plot. In the annotations, $r_{p}$ and $r_{s}$ denote Pearson and Spearman rank correlation coefficients; MAE is mean absolute error, RMSE is root mean square error, and SD is standard deviation. Point colours are as in Figure 5.

The 197 modified observations were classified using the anatomical-level narrative findings recorded during adjudication (Figure 5C). Vessel over-detection was most frequent (*n* = 97; mean automated-minus-expert difference +2.7 points); the expert score was lower than the automated score in 95 of 97 observations. Missed fibrosis accounted for 46 observations (mean difference—3.3 points), with a higher expert score in all 46. The 50 observations in the ‘other’ category had a mean automated-minus-expert difference of +2.0 points and a mean absolute difference of 2.84 points; the expert score was lower in 41 of 50. The four mixed cases were bidirectional, with a mean difference of 0.0 points. Overall, the expert score was lower than the automated score in 138 observations (70.1%) and higher in 59 (29.9%), indicating that over-detection was the dominant direction of discrepancy. Agreement remained high in the pooled side-specific analyses (right lung Pearson r = 0.991, MAE 0.47; left lung r = 0.986, MAE 0.57; Figure 6), but these values were also inflated by unchanged pairs. The near-zero pooled bias (+0.18) reflects net cancellation of discrepancies in opposite directions and should not be interpreted as evidence of an unbiased detector.

These findings are exploratory. The Fibrosis Index and spatial scoring map were produced by the vessel- and structure-removal detector operating on 8-bit windowed images, which constrains separation of vessels from fibrosis (Section 2.5). Definitive validation will require independent blinded physician scoring and re-computation of the fibrosis masks from native Hounsfield-unit DICOM data. The present analysis is therefore interpreted as evidence of methodological feasibility rather than as a confirmatory clinical result.

**Figure 7:**
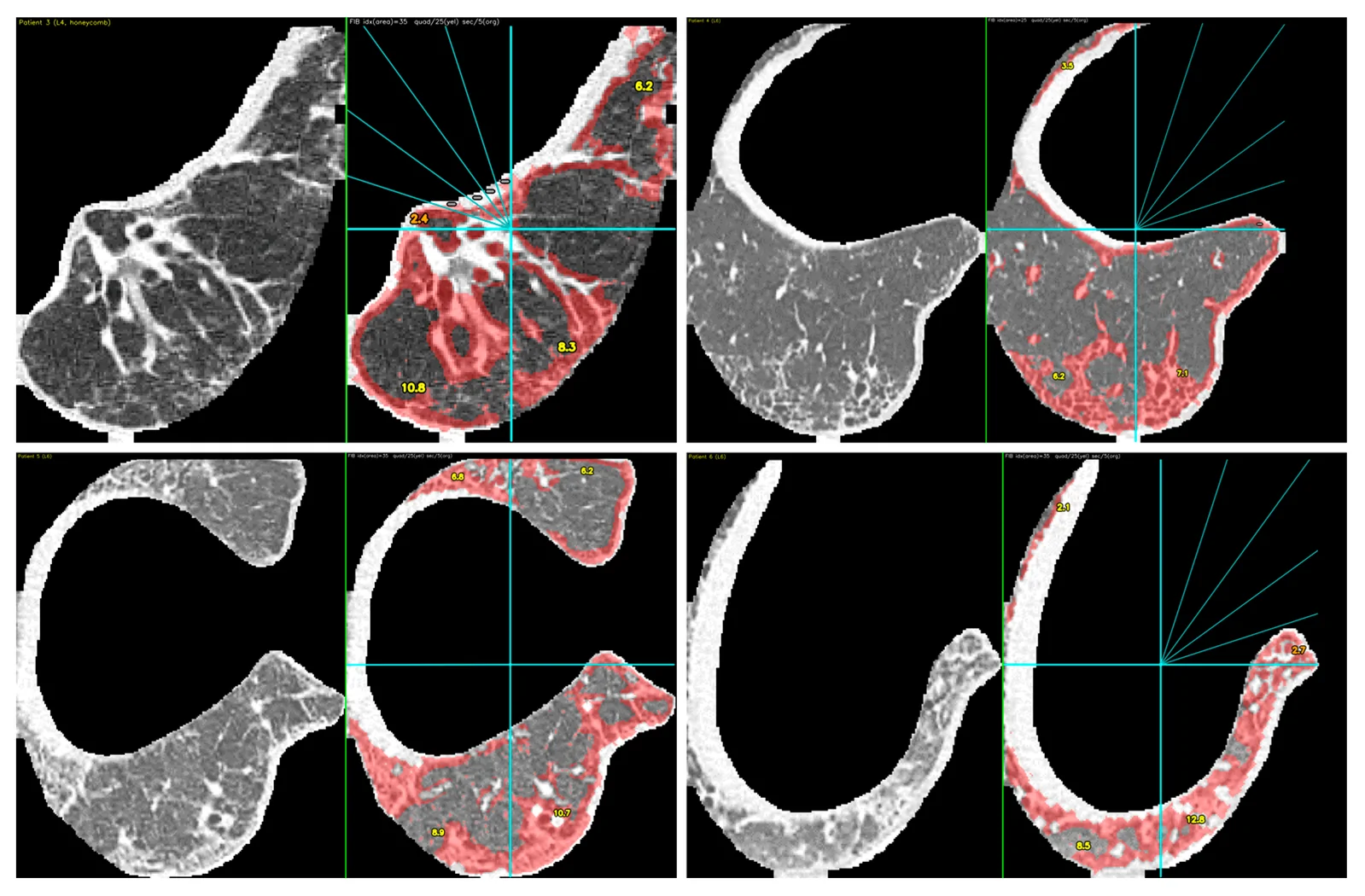
Representative per-lung dual scoring at selected anatomical levels in four cases from the agreement cohort, including a honeycomb-predominant lung (Patient 3). Each pair shows the input CT and scored map: after vessels and other non-fibrotic structures are removed, residual texture (red) is scored over radial quadrant/sector regions. In the embedded labels, ‘FIB idx(area)’ denotes the auxiliary per-level, per-lung area-weighted Fibrosis Index of Equation (3); v is a fixed label and does not denote volume. ‘quad/25 (yel)’ and ‘sec/5 (org)’ denote quadrant scores out of 25 in yellow and sector scores out of 5 in orange. The cases illustrate a range of fibrotic involvement and patterns. The detector is a prototype operating on 8-bit windowed images; pixel-level validation requires independently drawn fibrosis masks and implementation on native Hounsfield-unit data.

Figure 3 illustrates vessel- and structure-removal detection step by step in two representative lungs; Figure 7 presents per-lung dual scoring in four representative cases from the agreement cohort spanning the observed fibrosis range.

### 3.4. Angular Distribution, Read-Out Association, and Partition Sensitivity

The two read-outs of Section 2.7 and their dependence on the implemented partition were quantified on the same 1164 per-level, per-lung observations from 97 patients used for the agreement analysis, decoded through the production image path. This was a descriptive, implementation-focused analysis: it did not compare radial with rectangular or other partition schemes and did not test associations with clinical outcomes or events. All confidence intervals are patient-clustered bootstrap intervals (2000 resamples); no *p*-values are quoted because they underflow at this sample size.

#### 3.4.1. Involvement Is Not Angularly Uniform

**Figure 8:**
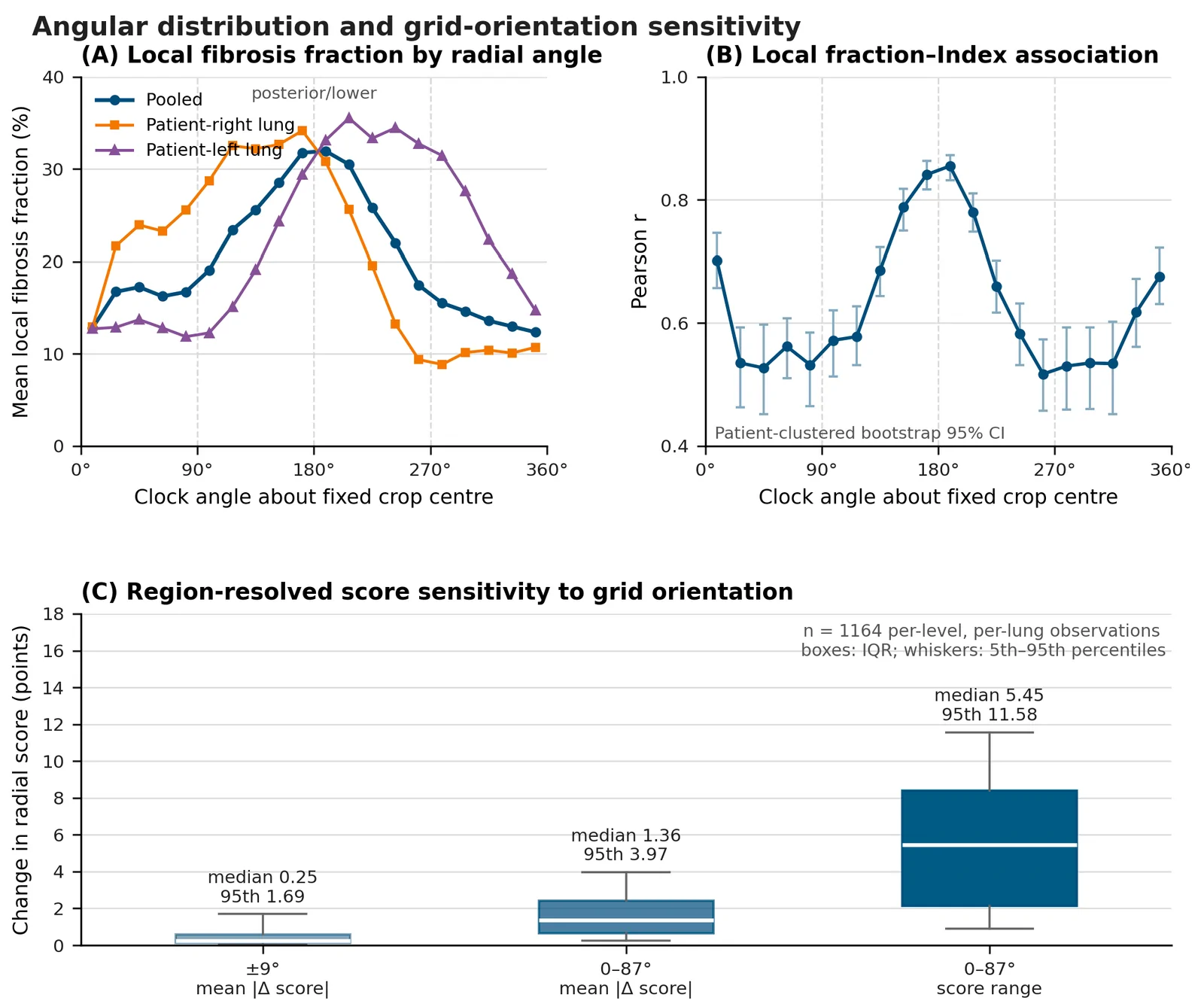
Angular distribution and grid-orientation sensitivity in the 97-patient agreement cohort. (**A**) Mean local fibrosis fraction in twenty 18° sectors about the fixed crop centre, shown pooled and separately for patient-right and patient-left lungs; 0° is the top/anterior direction and 180° the lower/posterior direction in the displayed crop. Sector counts varied from 714 to 1159 because sectors with lung occupancy ≤ one fifth were not evaluated; 1162 per-level, per-lung observations contained at least six evaluable sectors. (**B**) Pearson correlation between each sector’s local fibrosis fraction and the per-level, per-lung Fibrosis Index, with patient-clustered bootstrap 95% confidence intervals. (**C**) Distribution of changes in the region-resolved radial score under grid rotation across all 1164 observations; boxes show interquartile ranges and whiskers the 5th–95th percentiles. These plots describe angular heterogeneity and implementation sensitivity within this dataset; they do not compare partition schemes or establish clinical-outcome validity.

Across twenty 18° sectors around the crop centre, 1162 per-level, per-lung observations contained evaluable sectors. Mean fibrosis fraction ranged from 0.124 in the 342–360° sector to 0.320 in the 180–198° sector, a 2.59-fold posteriorly weighted gradient. Within an observation, the most- and least-involved quadrants differed by a median of 0.230 in fraction units; 987 of 1162 observations (85.0%) had at least a twofold difference. The most-involved quadrant was in the lower half of the displayed lung crop in 84.2% of observations. Side-specific analysis showed mirrored geometry: the patient-right lung most often peaked in its medial-lower quadrant (75.7% of 581 observations), and the patient-left lung in its corresponding medial-lower quadrant (73.3% of 581). Pooling the two sides obscures this mirror symmetry. These within-dataset angular distributions are shown in Figure 8A.

#### 3.4.2. How the Per-Level Fibrosis Index Relates to Angular Position

The association between each sector’s local fibrosis fraction and the per-level, per-lung Fibrosis Index varied by angle. Pearson r ranged from 0.517 in the 252–270° sector and 0.527 in the 36–54° sector to 0.855 in the 180–198° sector; regression slopes ranged from 0.0057 to 0.0131 per Index point. Figure 8B shows this angle-specific association. It indicates that a per-level scalar captures local involvement unevenly across angle, but it does not demonstrate that the selected radial partition is superior to another partition. The Index was also negatively associated with within-observation angular heterogeneity (r = −0.746; *n* = 1158), consistent with lower burden being more spatially patchy and higher burden being more diffuse. By definition, the Index contains no directional information.

#### 3.4.3. The Index Is Exactly Invariant to the Partition; The Radial Score Is Not

Sweeping grid orientation through its full 90° period and separately moving the partition origin from the fixed canvas centre to the lung centroid with ±3- and ±5-pixel jitter changed the Fibrosis Index by exactly 0.00 in all 1164 observations. This is an analytic property verified as an implementation check. The radial score was sensitive to partition placement. Under a small ±9° rotation, its median mean absolute change was 0.25 points. Across a full 90° sweep, the median mean absolute change was 1.36 points, the median worst-case range was 5.45 points (95th percentile 11.58), and the quadrant-subdivision decision changed in 674 of 1164 observations (57.9%). Moving the origin to the lung centroid changed the score by a median of 0.85 points (95th percentile 7.79) for a median displacement of 13.2 pixels. Figure 8C shows the distribution of radial-score changes under grid rotation.

For context, the full-sweep mean absolute sensitivity of 1.36 points was 0.44 times the MAE of the modified score subset (3.07) and 2.62 times the pooled MAE (0.52). This partition sensitivity does not alter the reported automated-versus-reference differences because the same fixed grid was used during non-blinded expert adjudication. It instead identifies a construct validity limitation: the absolute radial score depends partly on the selected grid orientation and origin. Without a comparator partition or clinical-outcome analysis, these results establish implementation sensitivity only.

#### 3.4.4. The Two Read-Outs Are Associated but Not Interchangeable

Across the 1164 per-level, per-lung observations, the Fibrosis Index averaged 22.7 (SD 12.5, range 0.0–61.6) and the radial score averaged 20.2 (SD 9.9, range 0.0–59.3). The two read-outs were strongly associated (Pearson r = 0.916, 95% CI 0.900–0.928; Spearman r = 0.943) but were not interchangeable: the ordinary least squares fit was radial score = 0.729 × Index + 3.64, and individual observations differed by as much as 30.9 points (mean absolute difference 3.22, median 1.02). Limits of agreement are not reported because the read-outs are intentionally different quantities. Exact algebraic decomposition attributed 95.5% of the mean absolute difference to equal angle versus lung pixel area weighting and 4.5% to forfeiture of sub-threshold regions. Even among 902 observations with no forfeited region, the mean absolute difference was 2.02 points. At least one quadrant was subdivided in 361 observations (31.0%). The difference between the read-outs is therefore a quantified consequence of their weighting schemes rather than an implementation inconsistency.

## 4. Discussion

In this study, we developed and evaluated an automated hybrid framework for CT-based quantification of pulmonary fibrosis. The approach integrates YOLO11n-seg lung segmentation with a standardised six-level anatomical protocol derived from the Taiwan ILD Semi-Quantitative Imaging Assessment Consensus [13]. The model achieved mIoU 0.926 ± 0.017 against physician-annotated ground truth on an internal holdout of five previously unseen patients (~0.95 in-sample during development). The small internal holdout warrants confirmation in a larger external cohort, and localisation and agreement were evaluated in separate analysis datasets.

Our work addresses variability in lung morphology across scans acquired at different inspiratory volumes. Non-rigid parenchymal deformation means that rigid or translational alignment cannot guarantee tissue-level correspondence within an axial plane. The standardised six-level protocol anchors sampling to anatomical landmarks and is intended to reduce inconsistency caused by inspiratory depth differences. This study did not evaluate longitudinal repeatability and therefore does not establish reproducibility across serial examinations. The consensus-derived protocol provides a standardised sampling framework for methodological evaluation.

The quantification stage was designed around tissue morphology rather than grey-level intensity, for the reasons set out in Section 2.5. Fixed intensity thresholds can suppress early subpleural fibrosis or retain vessels of similar brightness. The prototype therefore suppresses the continuous vascular tree while retaining residual irregular texture, with the aim of producing interpretable per-region estimates and preserving honeycombing. Its soft within-crop vertical-position and subpleural prior, multi-rule labelling, and final anatomical plausibility check address the fact that no single feature cleanly separates normal lung, vessels, pleura, and fibrosis. The present detector operates on 8-bit windowed images; use with native Hounsfield-unit data would require recalibration and separate validation and was not demonstrated here.

The area-weighted Fibrosis Index summarises per-level, per-lung burden and is mathematically independent of angular partitioning, whereas the radial score retains angular distribution but depends on grid placement. Section 3.4 and Figure 8 characterise both properties: the Index changed by 0.00 under all tested partition perturbations, while the radial score changed by a median of 0.25 points under ±9° rotation and by a median mean absolute 1.36 points across a full 90° sweep. The Index contains no directional information, and its association with local sector involvement varied by angle (r = 0.52–0.86). These results describe the behaviour of the implemented grid. Because no alternative partition and no clinical outcome association were evaluated, they cannot establish a scientific or clinical advantage for the radial scheme. Use of the same fixed grid for automated scoring and non-blinded expert adjudication means that partition choice does not explain the reported automated-reference differences, but it does not validate the partition itself.

The engineering benchmarks on an NVIDIA RTX A6000 separated model-only throughput from workflow timing. Peak YOLO11n-seg throughput was 1.48 ms/slice at batch size 512, but extrapolating this rate to a volume does not represent the implemented pipeline. In seven study patients, the sequential workflow required a mean of 22.5 s per patient, dominated by the two full-series localisation passes (19.839 s; 88.1%). All remaining stages together required approximately 2.67 s. Model loading and warm-up added 14.8 s once per session and were excluded from per-patient timing. Running the localisation passes as batched calls reduced that component to 3.48 s and projects a total of approximately 6.1 s per patient; the fully integrated batched workflow was not timed directly. For the downstream timing stages, six evenly spaced lung-bearing slices were used because the fitted production localisation coefficients were unavailable to the benchmark. This affects which slices were timed for segmentation, fibrosis detection, and scoring, but not the measured cost of the full localisation sweep.

The agreement analysis supports methodological feasibility and exploratory analytical concordance under non-blinded expert adjudication; it does not establish clinical validity. Of 1164 observations from 97 patients, 83.1% were unchanged after adjudication. Pooled association and agreement estimates were high, but the unchanged pairs lay on the identity line by construction. In the 197-observation modified-score subset, Pearson r was 0.918 and ICC(2,1) was 0.889, compared with pooled values of 0.989 and 0.988, respectively. The modified-score subset therefore provides the more informative summary. Confirmatory evaluation requires independently defined reference standards and multiple blinded readers.

### Limitations and Future Work

Several limitations define the scope of these findings. First, the internal segmentation holdout contained only 5 patients (845 slices), whereas the separate agreement analysis used 1164 observations from 97 patients; the larger agreement cohort does not substitute for external segmentation validation. Second, a single thoracic radiologist evaluated visible automated overlays and candidate values. This non-blinded design introduced anchoring risk, prevented estimation of inter-reader variability, and inflated pooled agreement through unchanged pairs. Discrepancy categories were derived from anatomical-level narrative findings rather than independently audited per-lung labels, and the 13 non-IPF ILD patients were not analysed separately. Third, the fibrosis detector uses 8-bit windowed images. Application to native Hounsfield-unit data requires recalibration of intensity-domain constants. No independently drawn pixel-level fibrosis masks were available, so fibrosis Dice or IoU could not be calculated; the per-lung adjudicated value also cannot validate regional boundaries or exclude cancellation of opposing regional errors. Overlay examination is more sensitive to discrete local errors than to smooth systematic boundary bias. Fourth, end-to-end timing was measured in only seven patients. The localisation sweep was measured in full, but downstream stages used evenly spaced lung-bearing slices because production localisation coefficients were unavailable; the approximately 6.1-s batched total is a projection rather than a directly timed integrated workflow. Fifth, the radial partition was not compared with rectangular or other partition schemes, and partition-derived scores were not tested against clinically important outcomes or events; the angular analyses therefore characterise within-dataset distribution and implementation sensitivity only. Future work requires native HU implementation, multicentre external cohorts, diagnosis-specific analysis, independently drawn fibrosis references, multiple blinded readers, and prespecified statistical comparison of alternative partitions against independent regional references and clinically important outcomes.

## 5. Conclusions

We developed an automated hybrid framework for side-resolved pulmonary fibrosis quantification on HRCT. YOLO11n-seg achieved an mIoU of 0.926 ± 0.017 in a small internal holdout of five previously unseen patients; the approximately 0.95 development value was in-sample. The six-level protocol provides anatomically anchored sampling, but the study did not evaluate longitudinal repeatability.

The per-level, per-lung radial score showed exploratory concordance under non-blinded expert adjudication: the expert and automated values were identical for 83.1% of 1164 observations, while ICC(2,1) in the 197-observation modified-score subset was 0.889. These findings support methodological feasibility only; they do not establish an imaging biomarker, an objective clinical decision-support tool, pixel-level fibrosis accuracy, blinded inter-reader agreement, an advantage over rectangular or other partition schemes, or clinical/prognostic validity of the radial score. Sequential end-to-end processing averaged 22.5 s per patient in seven study patients and was dominated by localisation; batching the localisation passes projects a total of approximately 6.1 s, but this integrated batched workflow was not timed directly. Larger external cohorts with independent blinded readers and independently drawn fibrosis references are required.

## Figures and Tables

**Table 1:** Baseline demographic, spirometry, gas-transfer, and recorded HRCT characteristics of the final 97-patient agreement cohort, stratified by descriptive anti-fibrotic treatment group.

	Nintedanib (*n* = 41)	Pirfenidone (*n* = 31)	No Anti-Fibrotic Therapy (*n* = 25)	*p*-Value
Age, years	76 (71–81)	73 (70–81)	78 (67–83)	0.324
Male sex, *n* (%)	28 (68.3)	20 (64.5)	19 (76.0)	0.646
Body height, cm	160.0 (155.9–164.5)	159.6 (150.7–164.6)	161.1 (154.7–166.7)	0.580
Body weight, kg	60.0 (54.4–66.6)	62.6 (51.3–68.5)	61.1 (54.3–67.1)	0.838
Body mass index, kg/m^2^	23.5 (20.8–25.6)	24.0 (22.7–26.0)	23.2 (20.8–26.5)	0.605
Current smoker, *n* (%)	1 (2.4)	2 (6.5)	1 (4.0)	0.817
COPD, *n* (%)	9 (22.0)	8 (25.8)	4 (16.0)	0.674
Cancer, *n* (%)	5 (12.2)	5 (16.1)	4 (16.0)	0.815
CAD, *n* (%)	7 (17.1)	4 (12.9)	4 (16.0)	0.938
D iagnosis				0.194
IPF, *n* (%)	33 (80.5)	27 (87.1)	24 (96.0)	
Other ILD, *n* (%)	8 (19.5)	4 (12.9)	1 (4.0)	
Spirometry and gas transfer				
FEV_1_, L	1.63 (1.30–2.07)	1.71 (1.34–2.01)	1.76 (1.56–2.19)	0.606
FEV_1_, % predicted	73.1 (64.0–81.0)	78.0 (71.2–90.0)	87.0 (70.0–101.0)	0.189
FVC, L	1.98 (1.67–2.63)	1.90 (1.63–2.50)	2.26 (1.67–2.86)	0.537
FVC, % predicted	70.0 (65.3–78.0)	75.0 (64.0–79.6)	83.0 (67.0–90.0)	0.324
DLCO, % predicted	46.9 (28.8–56.8)	52.0 (34.4–63.5)	51.0 (42.0–63.0)	0.530
Recorded HRCT pattern				0.237
UIP, *n* (%)	26 (63.4)	22 (71.0)	16 (64.0)	
NSIP, *n* (%)	8 (19.5)	5 (16.1)	1 (4.0)	
Bronchiectasis, *n* (%)	7 (17.1)	4 (12.9)	8 (32.0)	

Continuous variables are median (interquartile range); categorical variables are *n* (%). Available-case denominators were: age, 97; height, weight, and BMI, 94; FEV_1_ and FEV_1_ % predicted, 88; FVC, 73; FVC % predicted, 74; and DLCO, 46. Continuous variables used Kruskal–Wallis tests; categorical variables used Pearson χ^2^ or Fisher–Freeman–Halton exact tests as appropriate. Treatment groups were descriptive only. BMI: body mass index; COPD: chronic obstructive pulmonary disease; CAD: coronary artery disease; FEV_1_: forced expiratory volume in 1 s; FVC: forced vital capacity; DLCO: diffusing capacity for carbon monoxide. HRCT: high-resolution computed tomography; IPF: idiopathic pulmonary fibrosis; ILD: interstitial lung disease; UIP: usual interstitial pneumonia; NSIP: nonspecific interstitial pneumonia.

**Table 3:** Computational efficiency and hardware resource utilisation. BS: batch size; M: million parameters; GFLOPs: billion floating-point operations; I/O: input/output; ms: milliseconds.

Metric	Condition	Value (YOLO11n-Seg)
T raining Efficiency	Hardware	NVIDIA RTX A6000 (48 GB)
	Total Training Set Size	3315 slices (92 patients)
	Total Training Duration	~35 min
Inference Latency	Batch Size = 1	73.8 ± 9.7 ms/slice
Inference Throughput	Batch Size = 16	7.10 ms/slice
	Batch Size = 64	2.38 ms/slice
	Batch Size = 512 (Max)	1.48 ms/slice
Peak-throughput extrapolation (model only)	80 slices at BS = 512	~0.12 s; excludes I/O, localisation, and post-processing and is superseded by direct workflow timing
Model Complexity	Parameters	2.84 M
	Computational Load	10.4 GFLOPs
Workflow Timing	Measured sequential workflow (7 patients)	22.5 s/patient (median 24.0; range 17.3–24.9)

**Table 4:** Technical comparison of the proposed YOLO11n-seg vs. alternative architectures.

Architecture	mIoU (Full Training Set)	Training Time (Mins)	Params (M)	GFLOPs	Latency (ms/Slice, BS = 1) *	Peak Throughput (ms/Slice, BS = 512) *
YOLO11n-seg (Proposed)	0.95	~35	2.84	10.4	73.8 ± 9.7	1.48
U-Net	0.95	~70	~31.0	~150.0	>150.0	>45.0
Ladder-Net	0.95	~70	~15.2	~180.0	>180.0	>50.0
BT-Unet	0.85	~420	~31.0	~160.0	>200.0	>80.0
Mask R-CNN	0.85	~1400	~63.1	~300.0	>250.0	>120.0

* Note: Inference metrics (latency and peak throughput) were evaluated on the internal holdout using an NVIDIA RTX A6000 GPU. mIoU: mean intersection over union; BS: batch size; M: million parameters; GFLOPs: billion floating-point operations; ms: milliseconds.

**Table 5:** Ten-fold cross-validation. This table presents the mean intersection over union (mIoU) and standard deviation (SD) for the YOLO11n-Seg model.

Model	Mean ± Std. Dev.
YOLO11n-Seg	0.9402 ± 0.0044

## Data Availability

Paper-related scripts and usage instructions will be made available at https://github.com/zhirentsai/lung_seg (accessed on 1 September 2026). Patient data, CT images, derived patient-level datasets, model weights or checkpoints, and manuscript files will not be uploaded. De-identified supporting data may be requested from the corresponding author, subject to institutional approval and a data-use agreement.

## Appendix A. Vessel- and Non-Fibrotic-Structure Removal: Complete Parameter Specification

This appendix specifies every threshold, kernel, connectivity rule, and rule-application order used by the fibrosis-detection stage, so that the procedure can be reproduced. The values are the production values used for all results in this paper. The stage takes a single-lung 8-bit crop g and its binary lung mask and returns a vessel mask and a fibrosis mask. Appendix A.11 states how the constants were chosen, and Appendix A.12 identifies which are tied to the 8-bit intensity domain.

### Appendix A.1. Notation and Intensity Domain

g is the 8-bit single-lung crop, windowed at width 1500/level −600 HU, so −1350 HU maps to 0 and +150 HU to 255 and one grey level corresponds to 5.88 HU. The binary mask is lung, and vals denotes the intra-lung values g[lung]. Connected-component labelling uses 8-connectivity and box filters use reflected borders. P65, P72 and P80 are the 65th, 72nd and 80th percentiles of vals.

### Appendix A.2. Air/Tissue Separation

Otsu’s threshold on vals as a 256-bin 8-bit histogram gives otsu; the tissue cut-off is air_cut = max(1, otsu − 8), the 8 grey-level offset corresponds to approximately 47 HU, and tissue is g ≥ air_cut inside the lung.

### Appendix A.3. Local Texture

s5 is the local standard deviation over a 5 × 5 box. smooth is s5 < 16 (≈94 HU); irregular is tissue with s5 ≥ 15 (≈88 HU); solid_uniform is the 1-iteration dilation of (g ≥ P65 and s5 < 13) intersected with the lung (13 ≈ 76 HU). A further map, tissue with s5 > 20, is computed but not used downstream.

### Appendix A.4. Multi-Scale Tubularity

A Hessian (Frangi-type) filter is applied at scales 1.0, 1.5, 2.0, and 3.0 px with beta = 0.5 and c = max(0.5 × max(S), 1 × 10^−6^); only bright ridges are retained. The response is normalised by its maximum to give Vn, and the tubular map is Vn > 0.12 intersected with the lung eroded by 3 iterations, the erosion removing spurious response along the pleural rim.

### Appendix A.5. Connectivity Vessel Tree Grown from the Hilum

Bright pixels (g ≥ P72) are closed with a 3 × 3 element for 1 iteration. A distance transform dt of the closed set defines thick as dt ≥ 0.45 × max(dt); every connected component containing at least one thick pixel is retained, their union being the vessel tree, and local calibre is dt restricted to the tree. The hilar seed region is calibre ≥ 0.6 × max(calibre) intersected with smooth, falling back to the calibre criterion alone and then to the whole lung; dh is the distance to its centroid normalised by the crop diagonal. The central trunk is tree ∩ smooth ∩ tubular with dh < 0.25, the tubularity requirement preventing consolidation blobs from being taken as trunk.

### Appendix A.6. Honeycombing Protection

Dark holes are connected components of (g < air_cut) inside the lung with area 2–40 px. Their local density hd is the mean of the hole mask over a 9 × 9 box. Wall contrast wc is the mean grey of a one-pixel dilated ring around the holes minus the mean grey inside them, both over an 11 × 11 box, clipped to [−50, 100]. Honeycombing is hd > 0.06 with wc > 30 (≈176 HU). A regional variant, irr_dens > 0.20 with irr_dens the mean of the irregular mask over an 11 × 11 box, captures a whole honeycomb patch including its internal cysts.

### Appendix A.7. Within-Crop Vertical-Position and Subpleural Prior

db is the distance transform to the lung boundary normalised to a maximum of 1, and yb is the normalised vertical image coordinate within the axial single-lung crop. The implementation variable P_basal = clip((yb − 0.45)/0.45, 0, 1) is retained here for reproducibility; geometrically, it is a two-dimensional vertical-image-position term rather than a true cranio-caudal basal-level coordinate. The subpleural term is P_sub = clip((0.30 − db)/0.30, 0, 1). They are combined as P_fib = 1 − (1 − P_basal)(1 − P_sub), followed by P_fib = clip(P_fib + 0.30 × P_basal × P_sub, 0, 1). Three operating points are used: 0.30, 0.40, and 0.45.

### Appendix A.8. Pleural Band and Solid-Structure Suppression

Within the outermost ring db < 0.10, the subset that is also g ≥ P65 and smooth is examined; connected components of size at least max(60 px, 0.12 × its total area) are taken as pleura and dilated once. Honeycomb walls have high s5, are therefore not smooth, and are not captured here. Solid structures are detected per connected component of g ≥ P65 with area A ≥ 8 px using convexity (convex-hull perimeter divided by perimeter), circularity (4πA/perimeter^2^) and su_frac (the fraction of the component that is solid_uniform). A component is accepted if su_frac ≥ 0.55 and convexity ≥ 0.82, capturing smooth banded or blocky structures, or if A < 0.015 × lung area and circularity ≥ 0.55, capturing small round vessel cross-sections too small to judge by texture. The result is dilated twice.

### Appendix A.9. Initial Labelling and Iterative Cross-Checking

With lung_inner, the lung eroded twice, the initial vessel candidate set is the union of (tree or tubular) with P_fib < 0.45, the central trunk, solid_uniform with P_fib < 0.45, and the solid structures, intersected with the lung and excluding honeycombing and the pleural band; the initial fibrosis candidate set is the union of honeycombing, honeycomb regions and (irregular and tissue), intersected with lung_inner and excluding the pleural band and solid structures. Fibrosis is labelled first and vessel second, so vessel takes precedence on overlap. Five rules are then applied in fixed order and iterated to a fixed point, to a maximum of 6 iterations. R1: a vessel must connect to the central trunk or lie centrally (P_fib < 0.30); an isolated vessel becomes fibrosis if irregular with P_fib ≥ 0.45 and normal otherwise. R2: fibrosis must be anchored at or connected to a region with P_fib ≥ 0.40, and becomes normal otherwise. R3: fibrosis that is smooth, uniform and central (P_fib < 0.45) becomes vessel. R4: honeycombing, honeycomb regions and peripheral irregular texture with P_fib ≥ 0.45, inside lung_inner and outside the pleural band and solid structures, are re-added as fibrosis if currently normal. R5: any fibrosis overlapping a solid structure becomes vessel, irrespective of position.

### Appendix A.10. Output Post-Processing and the Second Pass

The vessel mask is closed with a 3 × 3 element once and intersected with the lung outside the pleural band; vessel components larger than 0.02 × lung area whose tubular fraction is below 0.18 are released as more likely consolidation. The 3-iteration dilation of (g ≥ P80 and s5 < 13) can never be fibrosis. The fibrosis mask is therefore the fibrosis label minus the vessel mask, the pleural band and that exclusion, restricted to lung_inner. With ring the lung minus its 4-iteration erosion, any fibrosis component lying more than 70% inside that ring is removed as pleural or chest-wall band while subpleural fibrosis extending inwards is retained. A second pass then removes the central airway and vessel tree while protecting the subpleural band: with a bright percentile of 80, a band fraction of 0.18, a minimum trunk distance-transform value of 2.5 px and one dilation, the interior is defined by distance to the lung boundary exceeding max(3 px, 0.18 × maximum distance); the trunk is the interior bright pixel of maximum distance-transform value, and the connected bright component containing it, restricted to the interior and dilated once, is subtracted from the fibrosis mask. If no interior trunk reaches 2.5 px, nothing is removed.

### Appendix A.11. How the Parameters Were Chosen

These constants were set by visual iteration on development cases and were not obtained through a formal search or optimisation procedure. No independent per-pixel fibrosis reference mask was drawn in this study, so no labelled objective was available for such a search; the adjudicated reference was one semi-quantitative value per lung at each selected anatomical level, not a per-pixel or independently assessed per-region label. The displayed delineations were examined during non-blinded adjudication (Section 3.3). The configuration should therefore be interpreted as documented and reproducible, not as optimised, and the sensitivity of the output to each constant has not been characterised.

### Appendix A.12. Which Constants Depend on the 8-Bit Intensity Domain

For migration to Hounsfield units the constants separate cleanly. Domain-dependent are the uint8 Otsu computation and its −8 grey-level offset, the local-standard-deviation thresholds 16, 20, 15, and 13, and the wall-contrast threshold 30 with its clip range [−50, 100]; under the window used here these convert as HU = 5.882 × g − 1350 for absolute values and by a factor of 5.882 for differences and standard deviations. Domain-independent are all percentile ranks (65, 72, 80), the self-normalised tubularity operating point 0.12, and every geometric, area, or fraction constant, including 0.45, 0.30, 0.40, 0.55, 0.82, 0.25, 0.10, 0.20, 0.06, 0.18, and 0.70, the area limits 0.015 and 0.02 of the lung area, the hole-size window 2–40 px, and all iteration, kernel, and box sizes.
